# Phylogenomic Reconstruction Indicates Mitochondrial Ancestor Was an Energy Parasite

**DOI:** 10.1371/journal.pone.0110685

**Published:** 2014-10-15

**Authors:** Zhang Wang, Martin Wu

**Affiliations:** Department of Biology, University of Virginia, Charlottesville, Virginia, United States of America; University of Hull, United Kingdom

## Abstract

Reconstruction of mitochondrial ancestor has great impact on our understanding of the origin of mitochondria. Previous studies have largely focused on reconstructing the last common ancestor of all contemporary mitochondria (proto-mitochondria), but not on the more informative pre-mitochondria (the last common ancestor of mitochondria and their alphaproteobacterial sister clade). Using a phylogenomic approach and leveraging on the increased taxonomic sampling of alphaproteobacterial and eukaryotic genomes, we reconstructed the metabolisms of both proto-mitochondria and pre-mitochondria. Our reconstruction depicts a more streamlined proto-mitochondrion than these predicted by previous studies, and revealed several novel insights into the mitochondria-derived eukaryotic metabolisms including the lipid metabolism. Most strikingly, pre-mitochondrion was predicted to possess a plastid/parasite type of ATP/ADP translocase that imports ATP from the host, which posits pre-mitochondrion as an energy parasite that directly contrasts with the current role of mitochondria as the cell’s energy producer. In addition, pre-mitochondrion was predicted to encode a large number of flagellar genes and several cytochrome oxidases functioning under low oxygen level, strongly supporting the previous finding that the mitochondrial ancestor was likely motile and capable of oxidative phosphorylation under microoxic condition.

## Introduction

Mitochondria are eukaryotic organelles with a bacterial origin. Known as the endosymbiotic theory, it is now widely accepted that mitochondria originated once from an alphaproteobacterium probably two billion years ago [1]. However, it remains unclear what constituted the initial endosymbiosis between the alphaproteobacterium and its host [2], [3], [4]. Specifically, what was the role played by the mitochondrial ancestor that initiated the endosymbiosis? Were mitochondria originated under oxic, microoxic, or anoxic condition? Did mitochondria arise at the same time as, or subsequent to, the appearance of nucleus? What was the driving force behind the initial symbiosis [4], [5]? Several hypotheses have been proposed to account for circumstances of the founding endosymbiotic events [4], [6], [7], [8]. The “hydrogen hypothesis” proposes the metabolic syntrophy between a H_2_-producing alphaproteobacterium and a H_2_-dependent archaeon as the driving force behind the endosymbiosis [4]. This hypothesis allows the possibility of a simultaneous origination of mitochondrion and nucleus, with the same alphaproteobacterium also contributing to the rise of nucleus by fusing its genome with the host genome [3]. Therefore, the “hydrogen hypothesis” directly challenges the traditional serial endosymbiosis model in which the host is posited to be a full-fledged, nucleus-containing (but amitochondriate) eukaryote. In contrast, the “oxygen scavenger” hypothesis proposes that the removal of the toxic oxygen by the alphaproteobacterium from the anaerobic host has driven the initial symbiosis [5]. The circumstances under which the founding events occurred remain highly debated.

Reconstructing the gene complement of mitochondrial ancestor can shine light on the origin of mitochondria. It will help us better understand the timing of its signature reductive evolution. More importantly, reconstruction of its metabolism will help elucidate the driving force of the endosymbiosis by testing the alternative hypotheses. For example, whether the initial endosymbiotic condition was aerobic or anaerobic is a key yet debated point among different hypotheses [4], [5]. The hydrogen hypothesis supports anaerobic syntrophy whereas the oxygen scavenger hypothesis supports aerobic mutualism. A recent study predicted the presence of both flagella and a cytochrome cbb3 oxidase in the mitochondrial ancestor and suggested that the mitochondrial ancestor was motile and capable of oxidative phosphorylation under microoxic condition [9]. However, this prediction was largely based on the analysis of one bacterial genome and needs to be further evaluated.

Although a key to understanding of the origin of mitochondria, mitochondrial ancestor reconstruction faces a multitude of problems. Firstly, there have been extreme genome reduction and dramatic metabolic turnover during mitochondria’s transformation from a bacterium to an organelle. For example, mitochondrion of *Reclinomonas americana*, the least derived mitochondrion recognized so far, encodes only 67 proteins in its genome [10]. Vast majority of genes of the ancestor of mitochondria have been either lost or transferred to the nucleus [3], resulting in the highly reduced genomes of modern mitochondria that only encode proteins functioning in translation and ATP production. To reconstruct the mitochondrial ancestor, these lost genes need to be recovered.

Secondly, a robust phylogenetic framework required for ancestral reconstruction has been lacking. Although mitochondria have been firmly placed within alphaproteobacteria, their exact phylogenetic position is uncertain. Weak phylogenetic signal and serious systematic errors such as long-branch attraction and sequence composition bias have all hampered the effort to pinpoint the origin of mitochondria. Nevertheless, recent phylogenomic studies with increased genomic sampling have started to form a consensus by placing mitochondria in or near the *Rickettsiales* order [2], [11], [12], [13], [14].

**Figure 1:**
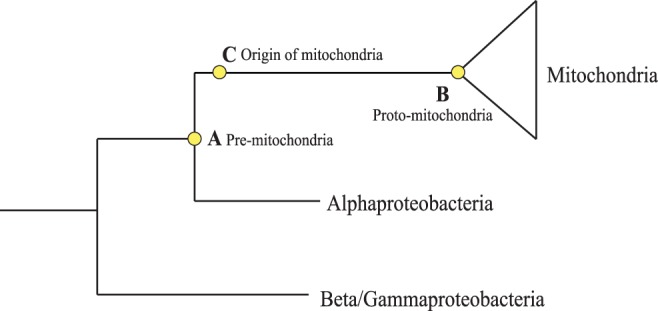
Key time points in mitochondria evolution. Time point A represents pre-mitochondria, the last common ancestor of mitochondria and alphaproteobacteria. Time point B represents proto-mitochondria, the last common ancestor of all contemporary mitochondria. Time point C represents the origin of mitochondria.

Previous studies have reconstructed the last common ancestor (LCA) of all mitochondria [15], [16]. Although insightful, results from these studies have serious limitations when it comes to understanding the origin of mitochondria. Figure 1 illustrates our point. Assuming the evolution of mitochondria is not reversible, point A represents the LCA of mitochondria and their alphaproteobacterial sister clade (for simplicity, hereafter referred as pre-mitochondria), while point B represents the LCA of all mitochondria (proto-mitochondria). Mitochondria emerged somewhere along the stem between points A and B, i.e., after they split off from alphaproteobacteria but before the divergence of the eukaryotic lineages (point C). All previous studies essentially reconstructed proto-mitochondria by simply pooling mitochondrial genes (including genes that have been transferred to the nucleus). Considering the dramatic transformation after the origin of mitochondria and the massive gene losses associated with this transformation, reconstructing proto-mitochondria would reveal little of what it looked like at the origin of mitochondria and therefore provide limited insights into the initial endosymbiosis event.

In order to understand what was happening at the origin of mitochondria, ideally we should reconstruct the ancestral state at time point C. It is difficult to delineate point C in the tree, however, because the origination event was not associated with any lineage diversification. If point B is an end point for studying the origin of mitochondria, then point A represents a good starting point. To gain better insights into the origin of mitochondria, it would be imperative for us to reconstruct pre-mitochondria at point A.

## Results and Discussion

### Identifying mitochondria-derived nuclear genes

Recovering mitochondrial genes that were lost to nucleus (hereafter referred as mitochondria-derived nuclear genes) is a prerequisite for mitochondrial ancestor reconstruction. Previous studies were based on a rather limited availability of bacterial and eukaryotic genomes at the time of their studies [15], [16]. Leveraging on a substantially increased representation of eukaryotic and alphaproteobacterial genomes, we performed a phylogenomic analysis to systematically identify mitochondria-derived nuclear genes. Eukaryotic genes with a top mitochondria/alphaproteobacteria BLAST hit were first clustered into gene families. A phylogenetic tree was reconstructed for each family and nuclear genes that clustered with alphaproteobacteria in the trees were identified as mitochondria-derived. Starting with 427,186 genes from 30 eukaryotic genomes representing a broad range of phylogenetic diversity, we identified 4,459 genes belonging to 394 families as mitochondria-derived nuclear genes. To eliminate recent, lineage-specific gene transfers between alphaproteobacteria and eukaryotes (e.g., between *Trichoplax adhaerens* and its rickettsial endosymbiont [17]), gene families were required to be present in at least two alphaproteobacterial and two eukaryotic lineages.

We estimated the specificity and sensitivity of our method following the procedure of [16]. For the false positive rate, we used the phylum of *Deinococcus/Thermus*, which has no close relationship with eukaryotic lineages, as a benchmark. Of all the 427,186 eukaryotic sequences we screened, only 278 sequences were clustered with *Deinococcus/Thermus* in the gene trees (false positive rate 0.07%), indicating our procedure has a very high specificity. To estimate the false negative rate, we used *Reclinomonas americana*, the hitherto least derived mitochondrial genome as the positive control. Considering that some of the *R. americana* mitochondrial genes have diverged too far to reliably identify their homologs, we used a subset of 50 *R. americana* mitochondrial genes that were present in at least 2 other mitochondrial genomes. 46 out of 50 genes were recovered by our procedure (false negative rate 8%), indicating that our procedure is also very sensitive.

### Reconstructing the metabolism of proto-mitochondria

**Figure 2:**
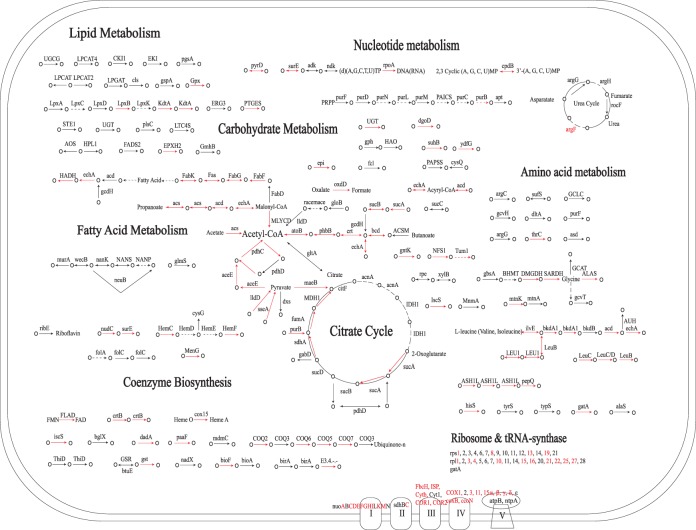
Reconstructed metabolism of proto-mitochondria. Black solid lines represent genes identified only in our reconstruction. Dotted lines represent missing genes in an otherwise complete pathway in our reconstruction. Red solid lines represent genes also present in Gabaldon et al. 2007.

**Figure 3:**
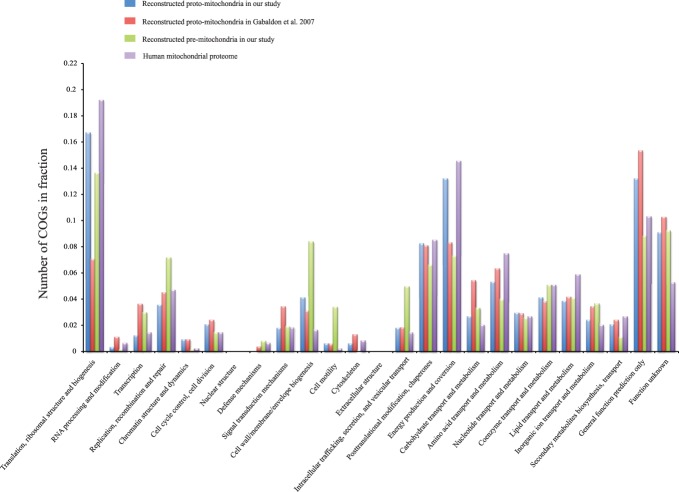
Function breakdown of reconstructed metabolisms of mitochondria ancestors. Within each class, from left to right are 1) the reconstructed proto-mitochondria in our study, 2) the reconstructed proto-mitochondria by Gabaldon et al. 2007, 3) the reconstructed pre-mitochondria in our study, 4) human mitochondrial proteome.

The 394 mitochondria-derived nuclear genes are presumably all present in proto-mitochondria. To reconstruct the metabolism of proto-mitochondria, we assigned 394 families to Clusters of Orthologous Groups (COGs) [18] and mapped them onto the Kyoto Encyclopedia of Genes and Genomes (KEGG) pathways [19] (Figure 2, Table S1). Pathways including pyruvate metabolism, TCA cycle, electron transport and ribosomal biogenesis, as well as fatty acids biosynthesis, beta-oxidation, branched-chain amino acids degradation (Leucine, Valine, Isoleucine) and the biosynthesis of ubiquinone, biotin and one carbon unit pool, were almost completely recovered, all of which are functional in modern mitochondria. Conversely, functional categories such as DNA replication and transcription were largely absent in our reconstructed metabolism, and the heterotrophic carbohydrate metabolisms such as glycolysis and pentose phosphate pathway were entirely missing. Therefore, our reconstruction depicts streamlined proto-mitochondria highly similar to modern mitochondria, while Gabaldon et al. study suggests an ancestor with more diverse functions (Figure 3) [16].

Compared to Gabaldon et al. study, our analysis is more sensitive. This is evident by the fact that despite reconstructing much leaner proto-mitochondria (our 394 vs. Gabaldon’s 842 gene families), we were able to fill many of the gaps in well-characterized mitochondrial pathways that were left open in Gabaldon’s reconstruction. For example, in the pyruvate metabolism and TCA cycle, we identified a pyruvate dehydrogenase E3 subunit (*lpdA/pdhD*, COG1249), a succinyl-CoA synthetase (*sucD*, COG0074) and a succinate dehydrogenase (*sdh2*, COG0479), which are essential for the pathways but were all missing in Gabaldon’s reconstruction. Similarly for ATP synthesis, we added one F0F1-type ATP synthase subunit (*atpC*, COG0355), 3 NADH dehydrogenases (*nuoB*, COG0377; *nuoM*, COG1008; *nuoL*, COG1009) and four cytochrome c components (*fbcC*, COG2857; *cyb561*, COG3038; COG3474; *cycM*, COG5274), completing a functional electron transport chain. For the assembly of iron-sulfur cluster, we added *iscU* (COG0822) and *iscA* (COG0820), two critical scaffold proteins upon which the cluster is assembled and transferred. In terms of the translation machinery, we identified 19 additional ribosomal proteins, along with 8 translation factors (*IF-2*, COG0532; *EF-P*, COG0231 and 6 GTPase (COG0012, COG0206, COG0050, COG2262, COG0218, COG1159)) and 3 aminoacyl-tRNA synthetases (COG0124, COG0162, COG0180). Also, we added a *COQ3* (COG2227) enzyme involved in the ubiquinone biosynthesis, essentially recovering a fully functional *de novo* ubiquinone biosynthesis pathway. In the biotin metabolism, we added a biotin-protein ligase (*birA*, COG4285), the key enzyme that connects the biotin metabolism with fatty acid biosynthesis.

Our reconstruction also has higher specificity. Of the 156 gene families we identified that are present in human nuclear genome, 104 (66.7%) families have been identified in the human mitochondrial proteome, compared with 121 out of 355 (34.1%) families in Gabaldon et al. 2007. Similar results were also observed in yeast and Arabidopsis (Table 1). In addition, 56.1% (221 out of 394) of the families identified in our study contain at least one gene with a N-terminal mitochondria targeting signal, compared to 30.7% (258 out of 842) in Gabaldon et al. 2007. Since mitochondria-derived nuclear genes are often recruited back to mitochondria, the higher percentage of mitochondria-localized nuclear gene families in our reconstruction indicates higher specificity of our results.

**Table 1:** Comparison between proto-mitochondrial reconstructions of this study and Gabaldon et al. 2007.

		This study	Gabaldon et al. 2007
Number of genomes	Total	1613	144
	Alphaproteobacteria/*Rickettsiales*	171/67	11/2
	Eukaryotes	30	16
Nuclear gene families in mitochondria proteome	Human	66.7%	34.1%
	Yeast	69.5%	46.8%
	Arabidopsis	63.3%	42.6%
Gene families with mitochondrial-targeted signal		56.1%	30.7%

The reasons for the increased sensitivity and specificity of our results could be at least twofold. First, our phylogenomic analysis used a much larger genome dataset representing a substantially broader range of taxon sampling (1,613 genomes in our study compared to 144 in Gabaldon’s 2007 study, Table 1). In particular, many more genomes were included for the *Rickettsiales* order that has shown close relationship to mitochondria (67 in our study compared to 2 in Gabaldon’s 2007 study, Table 1). The phylogenetic diversity of eukaryotic genomes was also greatly increased in our sampling, including 6 novel phyla that had not been sampled in previous studies (Figure S1) [15], [16]. Having a broader taxon sampling improved the power of phylogenetic analysis, which enabled us to more reliably trace the evolutionary history of gene families. Second, in Gabaldon study, nuclear genes that clustered with beta- and gammaproteobacteria were also identified as mitochondria-derived nuclear genes. The rationale is to increase the recovery rate of the *R. americana* mitochondrial genes as most of the ribosomal proteins were found to cluster with beta- and gammaproteobacteria in their phylogenetic analyses [16]. However, with increased alphaproteobacterial sampling we only identified two *R. americana* ribosomal proteins with such spurious patterns. Therefore, we think the criterion used in Gabaldon study is unnecessarily relaxed and could increase the false positive rate, leading to an overestimate of the number of mitochondria-derived nuclear genes. For example, genes involved in the pentose phosphate pathway all clustered with gammaproteobacteria or a mixture of gamma- and alphaproteobacteria and were identified as mitochondria-derived in Gabaldon study [16].

### Novel insights into mitochondria-derived eukaryotic metabolisms

Our reconstruction provides several novel insights into mitochondria-derived eukaryotic metabolisms. Of particular interest are a number of genes involved in the eukaryotic lipid metabolism (Table 2). For example, we identified 3 enzymes involved in steroid biosynthesis, including a squalene/phytoene synthase (COG1562), a sterol-C5-desaturase (COG3000) and a 1-deoxy-D-xylulose-5-phosphate synthase (COG1154) as mitochondria-derived, suggesting that mitochondrial ancestor has contributed to eukaryotic steroid biosynthesis. Mitochondrial origin of these enzymes is supported by functional studies. Mitochondria are known to play an essential role in the biosynthesis of steroids by providing sites for the onset of the process [20]. In return, steroids are also critical in maintaining the mitochondrial morphology [21]. Indeed, studies in *C. neoformans* and *T. brucei* indicated that mutants of squalene synthase and sterol desaturase were defective in mitochondrial membrane integrity [22], [23].

**Table 2:** List of mitochondria-derived nuclear genes involved in eukaryotic lipid metabolism.

Gene family	COG	Description	Lipidmetabolized	Cellularlocalization	Identified in Gabaldonet al. 2007
Group_236	COG2867	cyclase/dehydrase	Oligoketide	Unknown	N
Group_267	COG1215	ceramide glucosyltransferase	Sphingolipid	ER (MAM)	Y
Group_268	COG1562	squalene/phytoene synthase	Cholesterol	ER	Y
Group_946	COG1154	1-deoxy-D-xylulose-5-phosphate synthase	Terpernoid	ER	Y
Group_1713	COG3660	hypothetical protein	Likely lipid A	Mitochondria	Y
Group_1971	COG5597	sqdD glycosyl transferase	Sulfolipid	Unknown	N
Group_2416	COG1044	lpxD UDP-3-O- 3-hydroxymyristoyl glucosamine N-acyltransferase	Lipid A	Mitochondria	N
Group_2710	COG4689	acetoacetate decarboxylase	Ketone body	Unknown	N
Group_3620	COG1043	lpxA UDP-N-acetylglucosamine acyltransferase	Lipid A	Mitochondria	N
Group_3864	COG3000	Sterol C5 desaturase	Sterol	ER	N
Group_4604	COG1519	kdtA 3-deoxy-D-manno-octulosonic-acid transferase	Lipid A	Mitochondria	Y
Group_4794	COG0763	lpxB lipid-A-disaccharide synthase	Lipid A	Mitochondria	N

In addition, we identified a ceramide glycosyltransferase (COG1215) involved in the glycosphingolipids biosynthesis, carrying out ceramides glycosylation reactions. Interestingly, this enzyme is located at the “mitochondria-associated membrane”, a specific ER subdomain that bridges between ER and mitochondria [24]. Both glycosphingolipids and ceramides are ubiquitously present as essential membrane components in almost all eukaryotic cells and mitochondria, but are rarely identified in bacteria. Accordingly, the substrates and glycolipid products of the bacterial and eukaryotic glycosyltransferases were thought to be very different [25]. Therefore, the bacterial origin of this gene indicates acquisition of novel function by eukaryotes for synthesizing its own endomembranes and for the crosstalk and lipid trafficking between mitochondria and ER.

Our results also shed light on the mitochondria’s contribution to other eukaryotic metabolisms. For instance, we identified several genes (*purD*, COG0151; *purM*, COG0150; *mutT,* COG1051; *pyrD,* COG0167) involved in the *de novo* nucleotide biosynthesis as mitochondria-derived. Both *purD* and *purM* belong to the family of glycinamide ribonucleotide transformylase and catalyze different steps in the *de novo* purine biosynthesis. Mitochondria also contribute to the cytosolic purine biosynthesis by providing formate as the one-carbon unit. Consistently, the entire formate biosynthesis pathway was identified as mitochondria-derived in our results. On the other hand, *pyrD* is a mitochondria-localized protein critical for the pyrimidine biosynthesis [26]. *purD* and *purM* have been previously identified as of mitochondrial origin [13] but were missing in the results of Gabaldon et al. 2007. We also identified a number of genes (*glmS*, COG0449; *wecB*, COG1940; *neuB*, COG2089; *murA*, COG0766) involved in UDP-sugar biosynthesis. UDP-sugar provides essential modifications to various target proteins such as nuclear pore proteins and cytoskeleton components [27]. Thus it is tempting to speculate that these mitochondria-derived genes might participate in controlling the activities of these eukaryote-specific complexes.

### Reconstructing the metabolism of pre-mitochondria

**Figure 4:**
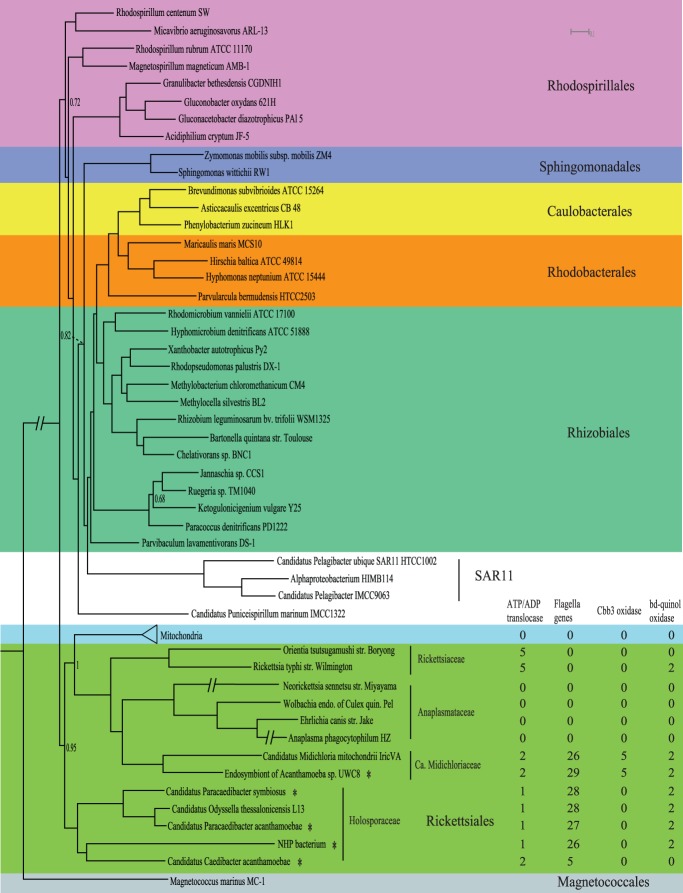
A species tree of alphaproteobacteria and mitochondria. The Bayesian consensus tree was based on concatenated protein sequences of 55 mitochondrial and nuclear genes. The tree was rooted using 8 beta- and gammaproteobacteria as the outgroup, as described in the methods. Asterisks indicate 5 newly sequenced *Rickettsiales* species. The posterior probability support values of the internal nodes are greater than 0.9 unless as indicated in the tree. Branches of several lineages are shortened for display purpose. The distribution of ATP/ADP translocase, flagella genes, cbb3 cytochrome oxidase and bd-quinol cytochrome oxidase in *Rickettsiales* and mitochondria is displayed besides the lineages.

To gain insights into the circumstances that surrounded the initial endosymbiosis event, it is imperative to reconstruct the metabolism of pre-mitochondria. To increase the power of ancestral reconstruction, we sequenced 5 novel linages of *Rickettsiales* (endosymbiont of acanthamoeba UWC8, *Candidatus* Caedibacter acanthamoebae, *Candidatus* Paracaedibacter acanthamoebae, *Candidatus* Paracaedibacter symbiosus, NHP bacterium) [28], [29]. These newly sequenced *Rickettsiales* species, together with 44 other alphaproteobacteria and 6 eukaryote representatives were used to reconstruct a Bayesian tree based on the concatenated protein sequence alignment of 55 mitochondrial and nuclear marker genes (Figure 4, hereafter referred as species tree). Mitochondria were placed within the *Rickettsiales* order, as a sister clade to the *Rickettsiaceae*, *Anaplasmataceae* and *Candidatus* Midichloriaceae families, all subtended by the *Holosporaceae* family. We note that the 5 newly sequenced *Rickettsiales* are all closely related to mitochondria. In particular, the 4 *Holosporaceae* species, together with previously sequenced *Candidatus* Odyssella thessalonicensis form an outgroup to the LCA of mitochondria and the other *Rickettsiales*, and therefore are extremely informative for reconstructing the ancestral state of pre-mitochondria.

**Figure 5:**
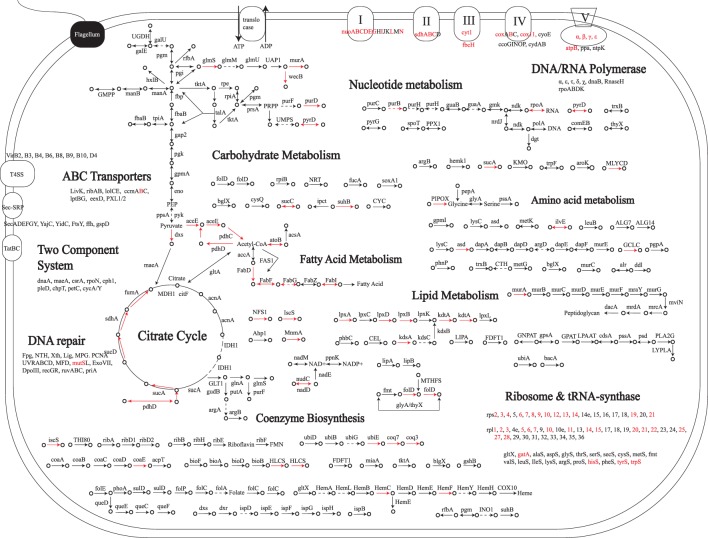
Reconstructed metabolism of pre-mitochondria. Black solid lines represent genes identified in our reconstruction while dotted lines represent missing genes in an otherwise complete pathway. Red lines represent genes present in proto-mitochondria in Figure 2.

Using tree in Figure 4 and a Bayesian character mapping method, we predicted the gene complement of pre-mitochondria to contain 887 COGs (Table S1). Based on the approximate linear relationships between the number of gene families, the number of genes and the genome size (Figure S2), we estimated the pre-mitochondrial genome to be 1.5–1.6 Mb, with 1100–1300 genes. This is typical of an obligate intracellular bacterium and suggests that genome reduction was well under way before mitochondria split from alphaproteobacteria, i.e., the origin of mitochondria. Figure 5 shows the reconstructed metabolism of pre-mitochondria. Compared to the highly specialized proto-mitochondria, pre-mitochondria were capable of much more diversified metabolisms. In addition to the major pathways involved in translation (13.6%), cell wall, LPS and membrane biogenesis (8.3%), energy production (7.2%), and DNA replication, recombination and repair (7.1%) (Figure 3), they were predicted to possess multiple key metabolic pathways including glycolysis, TCA cycle, pentose phosphate pathway, and fatty acid biosynthesis pathway. Also, pre-mitochondria possessed a large number of genes involved in synthesizing various cofactors, such as riboflavin, folate, biotin and ubiquinone. On the other hand, similar to most *Rickettsiales*, pre-mitochondria possessed a limited number of genes for synthesizing certain amino acids (Glutamine, Leucine, Valine and Isoleucine) from metabolic intermediates, but were deficient in *de novo* amino acid biosynthesis. Therefore pre-mitochondria had to obtain essential amino acids from the host. Accordingly, at least 5 amino acid transporters were predicted in pre-mitochondria.

Pre-mitochondria were predicted to lack most of the genes involved in the *de novo* nucleotide biosynthesis pathway, except for a few genes such as *purD* and *pyrD* that were also present in proto-mitochondria. Among *Rickettsiales*, the *de novo* nucleotide biosynthesis pathway is present in the family *Anaplasmataceae* but absent in all other lineages. Hence one interpretation is that the *de novo* nucleotide biosynthesis pathway was gained in *Anaplasmataceae*, as suggested by the Bayesian reconstruction method. Because of their isolated lifestyles and the lack of selection pressures for diversifying due to relatively constant intracellular environment, obligate intracellular bacteria such as *Anaplasmataceae* are thought to have limited opportunities for gene acquisition [30]. Therefore, we think acquiring the entire *de novo* nucleotide biosynthesis pathway, including 12 purine and 6 pyrimidine biosynthesis genes, is extremely unlikely in *Anaplasmataceae*. Instead, we think it is more likely that the nucleotide biosynthesis pathway was present in the ancestor of *Rickettsiales* and mitochondria and was subsequently lost multiple times in both *Rickettsiales* (except for *Anaplasmataceae*) and mitochondria.

### Pre-mitochondrion possessed flagella

**Figure 6:**
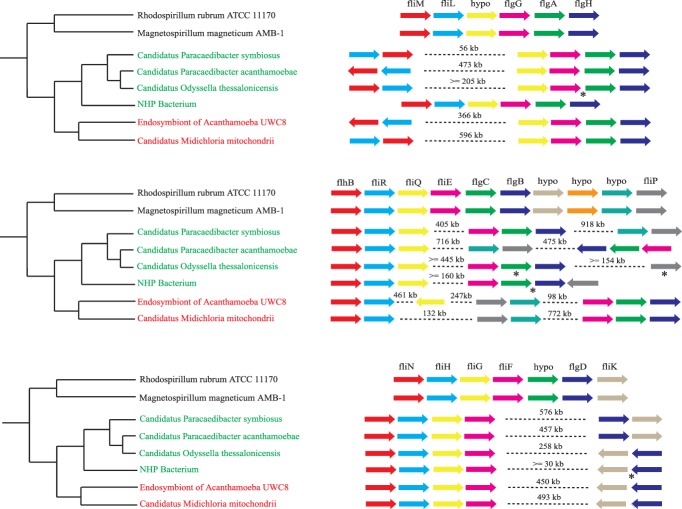
The synteny of flagellar genes. *Holosporaceae*, *Candidatus* Midichloriaceae, and free-living alphaproteobacteria representatives are highlighted in green, red and black respectively. Each arrow represents a gene in the cluster. Genome rearrangements are shown as dotted lines between two genes, with the distance between them shown above the lines. Because of the incomplete nature of some genome assemblies, the exact distance between two genes could not be determined. In this case, a minimum distance was estimated as the sum of distances of each gene to the end of the contig it was located in. For the same reason, the orientation of some genes could not be determined (indicated by asterisks below the genes).

A recent study has suggested that the mitochondrial ancestor possessed a flagellum [9]. However, this prediction was based on the presence of 26 flagellar genes in only one *Rickettsiales* species, *Candidatus* Midichloria mitochondrii. Interestingly, most of these 26 flagellar genes were found in 4 out of the 5 *Rickettsiales* endosymbionts we sequenced and also in the recently sequenced endosymbiont *Candidatus* Odyssella thessalonicensis (Figure 4). The phylogenetic tree of the concatenated flagellar genes is congruent with the *Rickettsiales* species tree, indicating that flagellar genes have been inherited vertically in *Rickettsiales* species. Consistently, they form syntenic gene clusters (Figure 6). It is therefore not surprising that pre-mitochondria were predicted to possess 25 COGs encoding the core flagellar components including the basal body, motor, hook, rod, filament and export apparatus [31]. Electron microscopy of the NHP bacterium has shown flagella at the basal end of its cell [32]. One recent study also observed flagella in two endosymbionts of Paramecium belonging to the *Lyticum* genus of the *Midichloriaceae* family [33]. Take together, these results strongly support the presence of flagella in pre-mitochondria.

### Pre-mitochondrion was capable of respiration at low oxygen condition

A key yet debated point among different hypotheses is whether the initial endosymbiotic condition was aerobic or anaerobic [4], [5]. Interestingly, it has been suggested that pre-mitochondria could respire at low oxygen condition because it possessed a cbb3-type cytochrome oxidase that mainly function under the microoxic condition [9]. Like the flagellar genes, this prediction was largely based on the presence of cbb3-type cytochrome oxidase in *Candidatus* Midichloria mitochondrii alone. Of the 5 *Rickettsiales* endosymbionts we sequenced, cbb3 oxidases were found only in the endosymbiont of acanthamoeba UWC8, the sister clade of *Candidatus* Midichloria mitochondrii. Although our reconstruction predicted the presence of cbb3 oxidase in the LCA of mitochondria and *Rickettsiales* (COG2010, COG2993, COG3278, COG0348, COG2217), the posterior probabilities were only 0.56. Therefore, we cannot exclude the alternative interpretation that cbb3 oxidases were absent in pre-mitochondria and were only gained in the *Candidatus Midichloriaceae* lineage. Interestingly, however, we also predicted the presence of a cytochrome bd-type quinol oxidase (COG1271, COG1294) in pre-mitochondria. The bd-type oxidases are widely distributed in the *Holosporaceae* and *Ca*. Midichloriaceae families (Figure 4), strongly suggesting that they were present in pre-mitochondria (posterior probability 0.89) and have been lost in mitochondria and other *Rickettsiales* lineages. Like cbb3-type oxidases, the bd-type oxidases are functional under limited oxygen level. Our study therefore provides strong and additional support to the hypothesis that pre-mitochondria were capable of oxidative phosphorylation under low oxygen condition.

### Pre-mitochondrion was an energy parasite

Intriguingly, pre-mitochondria were predicted to encode the plastid/parasite type of ATP/ADP translocase (COG3202; posterior probability 0.93), a hallmark protein of many obligate intracellular bacteria that is used to import ATP from the host, although remote homologs of this gene belonging to the major facilitator superfamily (MFS) transporters are also present in free-living bacteria [e.g., 34]. The ATP/ADP translocase commonly functions as an ATP/ADP antiporter that exchanges bacterial ADP for the host cell ATP [35]. In addition, it has been shown that some intracellular bacteria, including Chlamydia and Rickettsia, encode additional isoforms of this protein for the uptake of nucleotides to compensate for their inability to synthesize nucleotides *de novo* [36], [37]. Consistently, this gene family is absent in the *Anaplasmataceae* family of *Rickettsiales*, members of which all possess complete *de novo* nucleotide biosynthesis pathway [13], [38], [39]. Previous studies have suggested that there were ancient lateral gene transfers of this gene between the ancestors of *Chlamydiales*, *Rickettsiales* and plastids [35], [40] followed by largely vertical inheritance [41]. In consistence, our phylogenetic analysis shows that the gene tree is largely congruent with the species tree of the *Rickettsiales* order (Figure S3), suggesting that this gene has been vertically inherited in *Rickettsiales* and thus was most likely present in their last common ancestor and by inference, pre-mitochondria. If the nucleotide biosynthesis pathway was also present in pre-mitochondria as we have predicted, then this gene most likely functioned as an ATP/ADP exchanger instead of a nucleotide transporter in pre-mitochondria.

Remarkably, the plastid/parasite ATP/ADP translocase is evolutionarily unrelated to and functionally distinct from the ATP/ADP translocase in modern mitochondria, which exhibits an opposite polarity by exporting ATP into the host cytosol [42], [43], [44]. Therefore, our reconstruction posits pre-mitochondrion as an “energy scavenger” and suggests an energy parasitism between the endosymbiont and its host at the origin of mitochondria, as first proposed by Andersson et al. [5], [45]. This is in sharp contrast with the current role of mitochondria as the cell’s energy producer and contradicts the traditional endosymbiotic theory that the symbiosis was driven by the symbiont supplying the host ATP [46], [47]. The replacement of plastid/parasite ATP/ADP translocase by mitochondrial ATP/ADP translocase occurred subsequently, resulting in a reverse flow of ATP between mitochondrion and its host. This remarkable transformation in energy metabolism might mark the transition of mitochondria from a parasitic endosymbiont to a mutualistic organelle [48].

A recent systematic survey of bacterial symbiosis has shown that mutualisms can originate either directly from environmental free-living bacteria or from intracellular parasites [49]. A key difference between these two evolutionary routes is that to initiate symbiosis, free-living bacteria need to offer immediate benefits to the host while intracellular parasitic bacteria do not [50]. Our results suggest that mitochondria most likely originated from an obligate intracellular parasite and not from a free-living bacterium. This has important implications for our understanding of the origin of mitochondria. It implies that at the beginning of the endosymbiosis, the bacterial symbiont provided no benefits whatsoever to the host. Therefore we argue that the benefits proposed by various hypotheses (e.g., oxygen scavenger hypothesis and hydrogen hypothesis) are irrelevant in explaining the establishment of the initial symbiosis. Instead, it might be more appropriate to apply them to explain the transition of mitochondria from a parasite to a mutualistic organelle at a later stage.

## Materials and Methods

### Identification of mitochondria-derived nuclear genes

The phylogenetic distribution of all sequenced eukaryotic genomes was retrieved from GenomeOnline database (GOLD, http://genomesonline.org/). 30 eukaryotic genomes representing a broad range of eukaryotic diversity (10 phyla) were selected for identifying the mitochondria-derived nuclear genes (*Allomyce macrogynus, Arabidopsis thaliana, Batrachochytrium dedrobatidis, Caenorhabditis elegans, Chlamydomonas reinhardtii, Cryptococcus neoformans, Dictyostelium discoideum, Drosophila melanogaster, Encephalitozoon intestinalis, Entamoeba histolytica, Enterocytozoon bieneusi, Giardia lamblia, Homo sapiens, Leishmania major, Micromonas pusilla, Monosiga brevicollis, Naegleria gruberi, Nectria haematococca, Nematostella vectensis, Nosema ceranae, Phytophthora infestans, Plasmodium falciparum, Saccharomyce cerevisiae, Schistosoma mansoni, Spizellomyces punctatus, Strongylocentrotus purpuratus, Tetrahymena thermophila, Thalassiosira pseudonana, Trichoplax adhaerens, Trypanosoma brucei*).

For every single gene of 30 eukaryotic nuclear genomes, an initial BLASTP search was performed against 2,742 complete bacterial, archaeal, and mitochondrial genomes. A eukaryotic gene was retained for further phylogenetic analysis if its top five hits contained an alphaproteobacterial or mitochondrial sequence (e-value cutoff 1e-4). All eukaryotic genes passing the initial BLAST search were clustered into families using the Markov Cluster Algorithm [51]. Families that were present in at least two eukaryotic lineages were selected for phylogenetic analysis. For each retained protein family, its homologs from all complete bacterial genomes were retrieved by BLASTP search (e-value cutoff 1e-15). Protein sequences were aligned using MAFFT [52] and trimmed using ZORRO [53]. Phylogenetic trees were constructed using FastTree 2 [54]. When possible, each individual tree was rooted using three different rooting methods, rooting with Archaea or *Deinococcus* as the outgroup or midpoint rooting. Each of the rooted trees was scanned for a bipartition where eukaryotic genes clustered with alphaproteobacterial or mitochondrial homologs. A partition was retained as one gene family if it contained at least two eukaryotes and two alphaproteobacterial species. Paralogs, if existed in a family, were separated and each was treated as a new family of mitochondria-derived nuclear genes.

### Functional annotation of mitochondria-derived nuclear genes

Mitochondria-derived nuclear genes were classified into Clusters of Orthologous Groups (COGs) by hidden Markov model search using HMMer3 [55]. To reconstruct metabolic pathways, genes were mapped onto Kyoto Encyclopedia of Genes and Genomes (KEGG) database using KEGG Automatic Annotation Server (KAAS) [56] with “bi-directional best hit (BBH)” as the assignment method.

### Genome sequencing, assembly and annotation

4 endosymbionts of acanthamoebae (endosymbiont of acanthamoeba UWC8, *Candidatus* Caedibacter acanthamoebae, *Candidatus* Paracaedibacter acanthamoebae, *Candidatus* Paracaedibacter symbiosus) and one pathogen of shrimp NHP bacterium were sequenced by a combination of 454, Illumina and Pacific Biosciences SMRT sequencing. The genomes were assembled using Newbler 2.5.3, CLCGenomicWorkbench 6.0.1 or Celera assembler 8.2 and closed manually by resolving repeats using paired-end information and comparing assemblies using Mauve [57]. PCR and Sanger sequencing were used to close gaps between contigs when necessary. The genomes have been deposited at DDBJ/EMBL/GenBank as follows: endosymbiont of acanthamoeba UWC8 (CP004403), *Candidatus* Caedibacter acanthamoebae (CP008936-CP008940), *Candidatus* Paracaedibacter acanthamoebae (CP008941-CP008942), *Candidatus* Paracaedibacter symbiosus (JQAK00000000) and NHP bacterium (JQAJ00000000). Protein-coding genes were predicted using the GLIMMER software package [58].

### Alphaproteobacteria and mitochondria species tree

We selected a set of 49 alphaproteobacterial representatives using a tree-based greedy algorithm to maximize their phylogenetic diversity [59]. A set of 6 eukaryotic lineages (*Cryptococcus neoformans, Arabidopsis thaliana, Nematostella vectensis, Spizellomyces punctatus, Monosiga brevicollis, Phytophthora infestans*) were selected as eukaryotic representatives. Phyla *Alveolata, Amoebozoa, Euglenozoa* and *Diplomonadida* were not included because they had extremely long branches and therefore were prone to long branch attraction (LBA).

We selected a set of 26 mitochondria-encoded genes (*cob, cox2, cox3, nad1, nad2, nad3, nad4, nad4L, nad5, nad6, nad9, rpl2, rpl5, rpl6, rpl16, rps1, rps2, rps3, rps4, rps7, rps8, rps11, rps12, rps13, rps14, rps19*) and 29 mitochondria-derived nuclear genes (*cox11, sdhB, sucD, petA, erpA, hesB, ybjS, nuoC, nuoD, nuoF, nuoG, nuoI, rpl3, grpE, groEL, dnaK, clpB, clpP, hslV, engA, gidA, trmE, AFG1, apaG, bioC, hemN, ksgA, mraW, hypothetical*) as the marker genes for reconstructing the species tree. The list of mitochondria-encoded genes was taken from [13]. Three genes (*atp6, atp9, atpA*) were excluded from the original list because of their potential involvement in lateral gene transfer [60]. Three other genes (*cox1, yejR, yejU*) were excluded because of the potential paralogy problem. The nuclear genes were selected because they were single-copy genes and were distributed in at least two eukaryotic phyla. Protein sequences of 55 mitochondrial and nuclear marker genes from selected genomes were identified, aligned, trimmed and concatenated using AMPHORA2 [61]. From the concatenated protein sequence alignment, a Bayesian tree was reconstructed using PhyloBayes [62] with the -CAT -GTR options as recommended in the manual. Two independent MCMC chains were run and the chains were considered converged when the maxdiff dropped below 0.3, as suggested in the manual. The trees were sampled every 10 cycles and the beginning one fifth of the trees from each chain were discarded as burn-in. The tree was rooted using 8 beta- and gamma-proteobacteria as outgroup (*Nitrosomonas sp. Is79A3, Ralstonia solanacearum GMI1000, Dechloromonas aromatica RCB, Chromobacterium violaceum ATCC 12472, Francisella tularensis subsp. tularensis FSC198, Legionella pneumophila str. Lens, Escherichia coli str. K-12 substr. MG1655* and *Pseudomonas aeruginosa PA7*). The outgroup species were taken from a previous study [14]. We excluded *Buchnera* because it had a long branch and might cause the LBA artifact.

### Pre-mitochondria ancestral state reconstruction

Using the species tree, pre-mitochondria were reconstructed with a Bayesian character mapping inference algorithm implemented in BayesTraits V2 [63]. The Bayesian method has been shown to be superior to both the parsimony method and maximum likelihood method in accounting for uncertainties in both model parameters and phylogeny [63], [64]. The mitochondrial genes were compiled by combining the mitochondria-derived nuclear genes with the mitochondria-encoded genes. Mitochondrial and 148,485 genes of 49 alphaproteobacterial genomes were first assigned to 4,873 COGs by hidden Markov model search using HMMer3 [55]. Genes that could not be assigned to a COG were then clustered into families using the Markov Cluster Algorithm [51], creating 3,210 “expanded COGs”. The presence/absence of each COG in each species was treated as a binary trait and was used for the ancestral state reconstruction. Gamma distribution was adopted as the prior distribution with its parameter estimated from an initial maximum likelihood analysis. The “hyperprior” option was used to reduce the uncertainty in choosing priors in the MCMC. A total number of 1,050,000 iterations were performed, with the first 50,000 cycles discarded as burn-in. The average value of each binary state in the remaining 1,000,000 cycles was then taken as the probability of the presence of each COG in the reconstructed ancestral state.

## Supporting Information

Figure S1 **Overview of mitochondria-derived nuclear genes.** The eukaryotic species tree was reconstructed based on the concatenated 29 ribosomal proteins conserved among all three domains. Within each species, from innermost to outermost are the numbers of 1) nuclear genes, 2) mitochondrial genes, 3) mitochondria-derived nuclear genes, 4) mitochondria-derived nuclear gene families. The heights of the bars were scaled for display purposes. Each color in the tree represents a different eukaryotic phylum. 6 novel phyla that had not been sampled by previous studies were indicated by asterisks. Lineages highlighted in red represent amitochondriate eukaryotes. Branches with dots represent the ones with bootstrap support > = 80 (100 replicates).
(PDF)

Figure S2 **Correlation a) between the number of COGs and the number of genes, and b) between the number of COGs and the genome size of 49 alphaproteobacteria representatives.** In both graphs, the red dots represent pre-mitochondria.
(PDF)

Figure S3 **A maximum-likelihood tree inferred from amino acid sequences of the ATP/ADP translocase in** ***Chlamydiales*** **(blue),** ***Rickettsiales*** **(orange),** ***Bacteroidetes*** **(yellow) and plastids (green).** The tree was rooted by homologs in Microsporidia (*Encephalitozoon intestinalis, Encephalitozoon cuniculi, Enterocytozoon bieneusi* and *Nosema ceranae*). Branches of several lineages are shortened for display purpose. Bootstrap values (out of 100 replicates) are above 80 unless as indicated in the tree.
(PDF)

Table S1 **Gene content of proto-mitochondria and pre-mitochondria as COGs.**
(XLSX)
