# Supplementary figures and images for: Phylogenomic Reconstruction Indicates Mitochondrial Ancestor Was an Energy Parasite
Source: PLoS One. 2014 Oct 15;9(10):e110685. doi: 10.1371/journal.pone.0110685 (PMC4198247; doi:10.1371/journal.pone.0110685)

—0.1

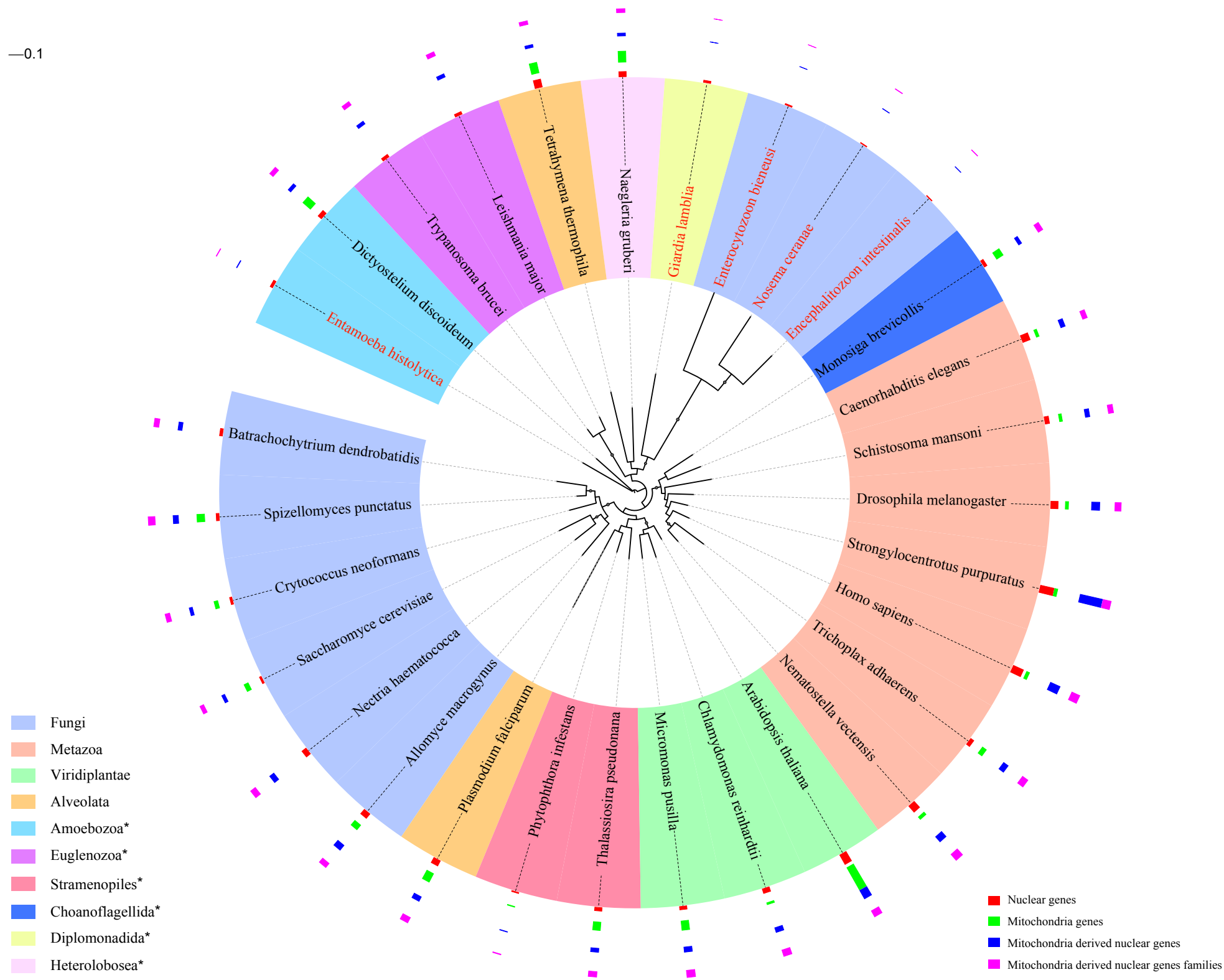

Supplement: Figure S1 — Overview of mitochondria-derived nuclear genes. The eukaryotic species tree was reconstructed based on the concatenated 29 ribosomal proteins conserved among all three domains. Within each species, from innermost to outermost are the numbers of 1) nuclear genes, 2) mitochondrial genes, 3) mitochondria-derived nuclear genes, 4) mitochondria-derived nuclear gene families. The heights of the bars were scaled for display purposes. Each color in the tree represents a different eukaryotic phylum. 6 novel phyla that had not been sampled by previous studies were indicated by asterisks. Lineages highlighted in red represent amitochondriate eukaryotes. Branches with dots represent the ones with bootstrap support > = 80 (100 replicates). (PDF) [file pone.0110685.s001.pdf]

a).

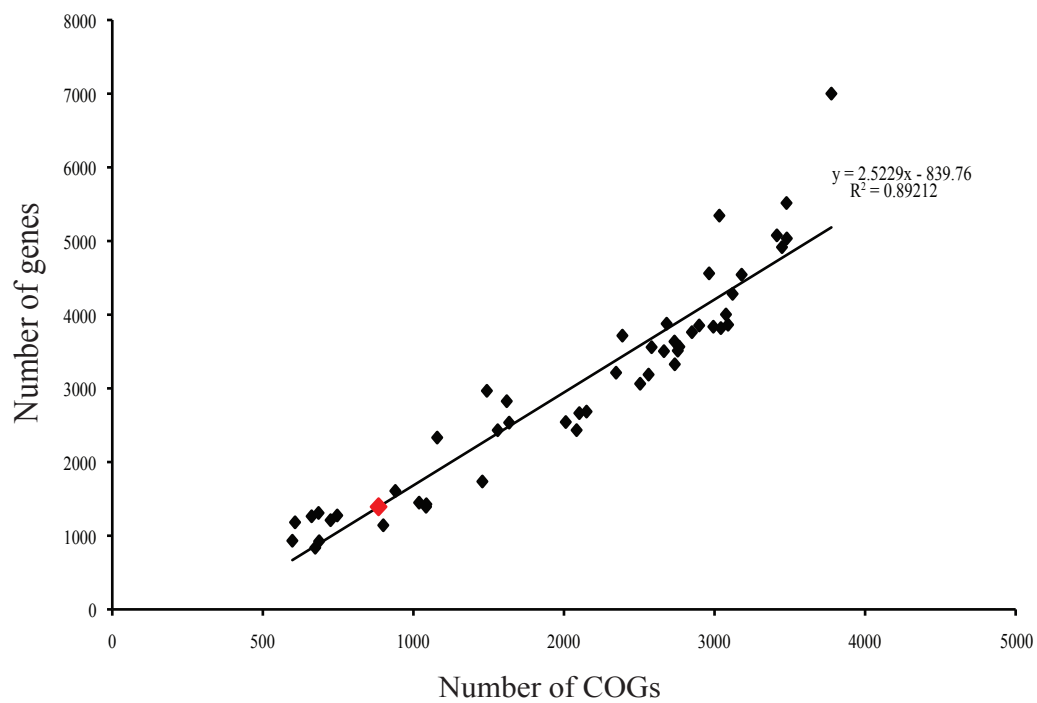

b).

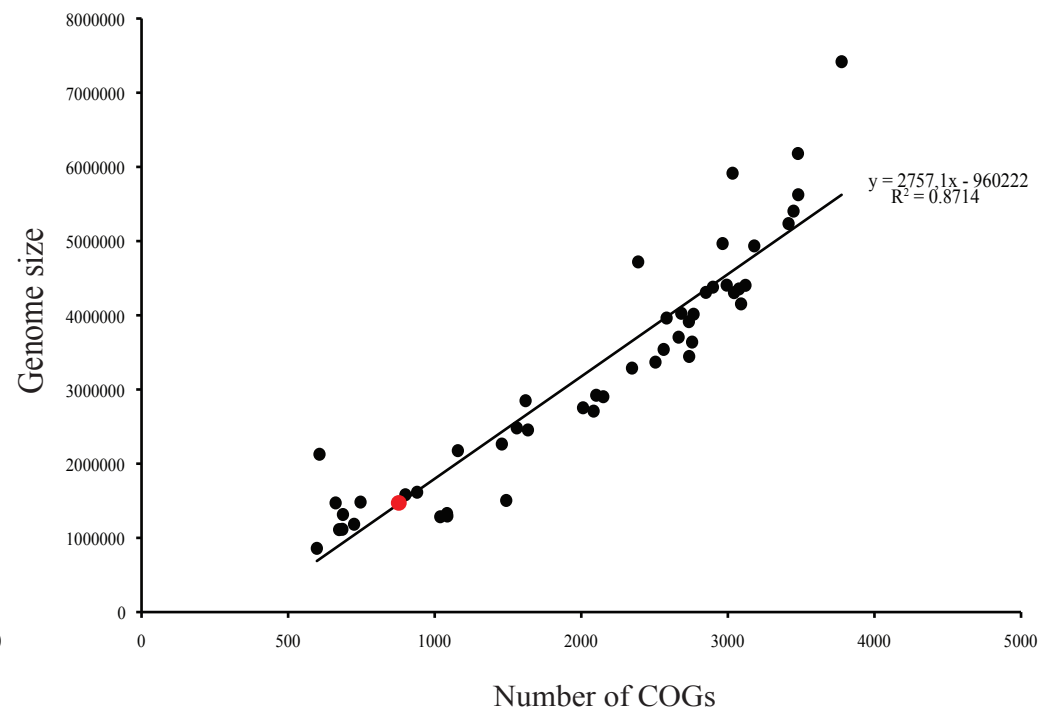

Supplement: Figure S2 — Correlation a) between the number of COGs and the number of genes, and b) between the number of COGs and the genome size of 49 alphaproteobacteria representatives. In both graphs, the red dots represent pre-mitochondria. (PDF) [file pone.0110685.s002.pdf]

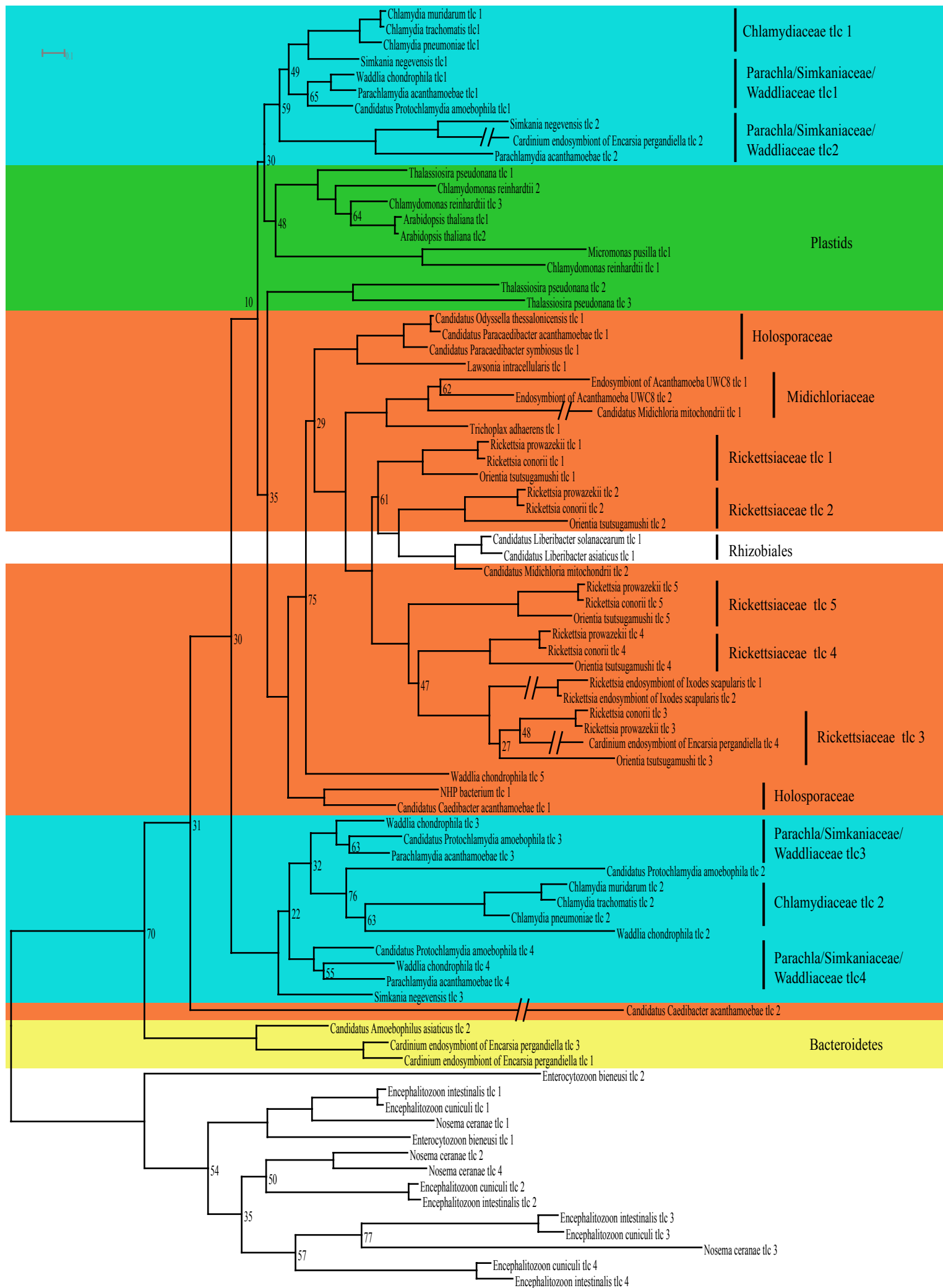

Supplement: Figure S3 — A maximum-likelihood tree inferred from amino acid sequences of the ATP/ADP translocase in Chlamydiales (blue), Rickettsiales (orange), Bacteroidetes (yellow) and plastids (green). The tree was rooted by homologs in Microsporidia (Encephalitozoon intestinalis, Encephalitozoon cuniculi, Enterocytozoon bieneusi and Nosema ceranae). Branches of several lineages are shortened for display purpose. Bootstrap values (out of 100 replicates) are above 80 unless as indicated in the tree. (PDF) [file pone.0110685.s003.pdf]
